# Loop-Mediated Isothermal Amplification Assay for Detecting Tumor Markers and Human Papillomavirus: Accuracy and Supplemental Diagnostic Value to Endovaginal MRI in Cervical Cancer

**DOI:** 10.3389/fonc.2021.747614

**Published:** 2021-11-01

**Authors:** Benjamin Wormald, Jesus Rodriguez-Manzano, Nicolas Moser, Ivana Pennisi, Thomas E. J. Ind, Katherine Vroobel, Ayoma Attygalle, Pantelis Georgiou, Nandita M. deSouza

**Affiliations:** ^1^ Cancer Research UK Cancer Imaging Centre, Division of Radiotherapy and Imaging, The Institute of Cancer Research, London, United Kingdom; ^2^ Department of Infectious Disease, Faculty of Medicine, Imperial College, London, United Kingdom; ^3^ Department of Electrical and Electronic Engineering, Faculty of Engineering, Imperial College London, London, United Kingdom; ^4^ Departmentof Surgical Oncology, Royal Marsden NHS Foundation Trust, London, United Kingdom; ^5^ Department of Histopathology, Royal Marsden NHS Foundation Trust, London, United Kingdom; ^6^ MRI Unit, Royal Marsden NHS Foundation Trust, Sutton, United Kingdom

**Keywords:** cervical cancer, loop mediated isothermal amplification, human papilloma virus, tumor markers, magnetic resonance imaging

## Abstract

**Objective:**

To establish the sensitivity and specificity of a human papillomavirus (HPV) and tumor marker DNA/mRNA assay for detecting cervical cancer that is transferrable to a Lab-on-a-chip platform and determine its diagnostic benefit in early stage disease when used in conjunction with high-resolution endovaginal magnetic resonance imaging (MRI).

**Methods:**

Forty-one patients (27 with Stage1 cervical cancer [Group1] and 14 non-cancer HPV negative controls [Group2]) had DNA and RNA extracted from cervical cytology swab samples. HPV16, HPV18, hTERT, TERC/GAPDH and MYC/GAPDH concentration was established using a loop mediated isothermal amplification (LAMP) assay. Thresholds for tumor marker detection for Group1 were set from Group2 analysis (any hTERT, TERC/GAPDH 3.12, MYC/GAPDH 0.155). Group 1 participants underwent endovaginal MRI. Sensitivity and specificity for cancer detection by LAMP and MRI individually and combined was documented by comparison to pathology.

**Results:**

Sensitivity and specificity for cancer detection was 68.8% and 77.8% if any tumor marker was positive regardless of HPV status (scenario1), and 93.8% and 55.8% if tumor marker or HPV were positive (scenario 2). Adding endovaginal MRI improved specificity to 88.9% in scenario 1 (sensitivity 68.8%) and to 77.8%% in scenario2 (sensitivity 93.8%).

**Conclusion:**

Specificity for cervical cancer detection using a LAMP assay is superior with tumor markers; low sensitivity is improved by HPV detection. Accuracy for early stage cervical cancer detection is optimal using a spatially multiplexed tumor marker/HPV LAMP assay together with endovaginal MRI.

## Introduction

Treatment of cervical intraepithelial neoplasia at colposcopy with cold knife cone (CKC) biopsy or large loop excision of the transformation zone (LLETZ) can sometime excise a small volume cervical cancer. Defining the presence and extent of any residual disease crucially determines subsequent surgical management (1). As women being treated for cervical precancer or early cancer are of similar age to women having their first child, fertility and reproductive effects of local excision of disease are important. The risk of post-surgical complications such as primary and secondary haemorrhage and cervical stenosis that may require further intervention, particularly where excisions are radical or repeated (2, 3), should be kept to a minimum. Increasing evidence suggests that the amount of cervical tissue excised or destroyed, measured as the cone length in excisional techniques, is a predictor for subsequent obstetric risk (4, 5). Moreover, a larger amount of residual cervical tissue detected on scan after treatment for both dysplasia and cancer is associated with improved obstetric outcomes (6–8). It is therefore critical to balance risk related to oncological *versus* reproductive outcomes by enabling the optimal local excisional treatment for these women.

Optimal surgical management may be achieved by assessing surgical margins on initial CKC or LLETZ biopsy supplemented by high-resolution endovaginal magnetic resonance imaging (MRI) (9). The latter offers a sensitivity of >90% for small tumors, albeit with a specificity around 70% for tumors <1.7cm^3^ because of confounding appearances from scarring and fibrosis after CKC biopsy or LLETZ (10). In these cases, detection of the human papillomavirus (HPV) genome (a key event in cervical carcinogenesis)and genetic tumor markers in cellular samples potentially provides an additional means of improving specificity of cancer detection prior to planning surgical management.

Of the 14 high-risk HPV types carcinogenic to humans, HPV-16 is consistently the most prevalent, detected in 60% of cases of cervical cancer (11). HPV-16 is detected more often in squamous cell carcinoma (62%) than in adenocarcinoma (50%), while HPV18 and HPV45 are detected more often in adenocarcinoma (32% and 12%, respectively) than in squamous cell carcinoma (8% and 5%, respectively). More than 50% of HPV16-positive and almost all HPV18-positive cases are associated with integration of virus genomes into cervical epithelial DNA (12, 13). Hybrid capture 2 (HC2; Digene Corporation, Gainthersburg, MD, USA) assays and polymerase chain reactions (PCR) for HPV detection amplify a broad spectrum of HPV genotypes and focus on the L1 gene but risk a false negative result because in cervical cancer this is lost in 10% of integrated HPV genomes (14). In these cases, detection of E6/E7 mRNA transcripts with PCR may be of higher prognostic value compared with HPV DNA testing (15).

As an alternative to PCR, loop-mediated isothermal amplification (LAMP) methods (16) incorporated into a lab-on-a-chip (LOC) allow rapid amplification of nucleic acids at a single temperature, typically between 63-65^0^C and have been used for the detection of infectious diseases such as malaria, dengue fever, bacterial and viral infections, notably SARS-CoV-2 (17–20). The lack of thermocycling makes this technique ideal for point-of-care testing. LAMP based assays have been successfully developed for a multitude of purposes, including genotyping HPV from cervical cytology samples (21, 22) but have not previously been combined with tumor markers associated with cervical cancer such as human telomerase reverse transcriptase (hTERT), which is significantly overexpressed in cervical lesions with low to nil expression in normal tissue and detectable in at least 90% of cervical cancers (23, 24), TERC and c-MYC, which are widely recognised tumor markers (25–28) for early detection of cancer. The aim of this work, therefore, was to establish the sensitivity and specificity of a HPV and tumor marker DNA/mRNA LAMP assay for detecting cervical cancer that is transferrable to a LOC platform and determine its diagnostic benefit in early stage disease when used in conjunction with high-resolution endovaginal MRI. HPV readouts from a conventional PCR platform (PCR) and cervical cytology/histology provided ground truth.

## Methods

### Study Design

A prospective pilot study (Molecular Diagnostics Using a novel Lab-on-a-chip and MRI for detecting cervical cancer, MODULAR, NCT03380741) was conducted in accordance with the Declaration of Helsinki (1964), and local research ethics committee and Health Research Authority (HRA) approval. Patients were studied in 2 groups: 1) new diagnosis of cervical cancer 2) non-cancer HPV negative controls. Written informed consent was obtained from each patient. In this hypothesis testing pilot study, where a biomarker could be positive or negative, and assuming the prevalence of a biomarker positive value (tumor DNA or HPV 16 or 18 DNA) among cancer patients is ~75% in women aged 30-39 years (29), we estimated that 24 patients with suspected cancer would establish the ability of the LAMP assay to detect cancer with a power of >0.9 at an alpha of <0.05, warranting a larger trial.

### Participants

Between August 2018 and May 2019, all patients with Stage 1 cervical cancer (squamous or adenocarcinoma on histology) referred for MRI to a tertiary oncology centre prior to being considered for curative surgery were invited to participate, so they formed a consecutive series of cases. Women with neuroendocrine tumors or unusual histological subtypes, or those unable to have MRI because of ferromagnetic implants or claustrophobia were excluded. A control group taken from women attending a separate local colposcopy clinic for follow up of either conservatively managed or previously treated cervical dysplasia, who were judged to have a normal cervix on colposcopic examination was recruited to establish threshold values for tumor marker positivity and confirm validity of a negative HPV result. As part of the routine management of the patients attending the colposcopy clinic for follow up, tests for both HPV 16 and 18 DNA (real time PCR using the GenoID assay kit) and HPV E6/7 mRNA (PreTect HPV-Proofer, Norchip) was obtained through The Doctors Laboratory (TDL). GenoID is a PCR based assay for the HPV L1 gene, followed by an ELISA based 96 well hybridisation assay to a cocktail of probes for the type-specific detection of high-risk HPV from 20 HPV phage types (30). PreTect Proofer is a real-time multiplex nucleic acid sequence based amplification assay for isothermal amplification of E6/E7 mRNA expressed by five high-risk HPV types (16, 18, 31, 33 and 45) using proprietary primer sets (31). These commercially available tests were considered the gold-standard (GenoID CE marked), and were performed in all 27 patients with cancer. HPV positivity on these tests therefore resulted in their exclusion from the control group. Patients who were positive for Type 45 and 31 on TDL HPV typing were not included in this comparative analysis.

### Cervical Swab Sampling

A cervical swab was taken either at an out-patient visit or prior to an examination under anesthesia (EUA) in all study subjects with cancer. In those patients with a normal cervix at colposcopy, the sample was taken as part of the colposcopic examination. Following insertion of a speculum, the cervix was swabbed with a standard cervical smear brush and the exfoliated cells deposited in PreservCyt transport medium. A separate, sequential cervical swab sample was examined conventionally to assess cytology and HPV DNA and RNA typing as per standard institutional clinical practice. Cytological sampling was adequate in all cases, so that inadequate sampling did not lead to withdrawal from the study in any instance.

### Sample Preparation

The exfoliated cells were pelleted and PreservCyt solution discarded. The remaining pellet was washed with phosphate buffered saline (PBS) solution and pelleted again. The PBS solution was discarded. DNA and RNA were extracted from the pellet using Qiagen AllPrep kit (Qiagen, Manchester, UK) according to the manufacturer’s instructions. Total DNA and RNA yield was determined using a NanoDrop ND-2000 spectrophotometer (Thermo Fisher Scientific, Waltham, USA). Only those samples which yielded both DNA and RNA were selected for analysis.

### Loop-Mediated Isothermal Amplification (LAMP) Assay

**Figure 1:**
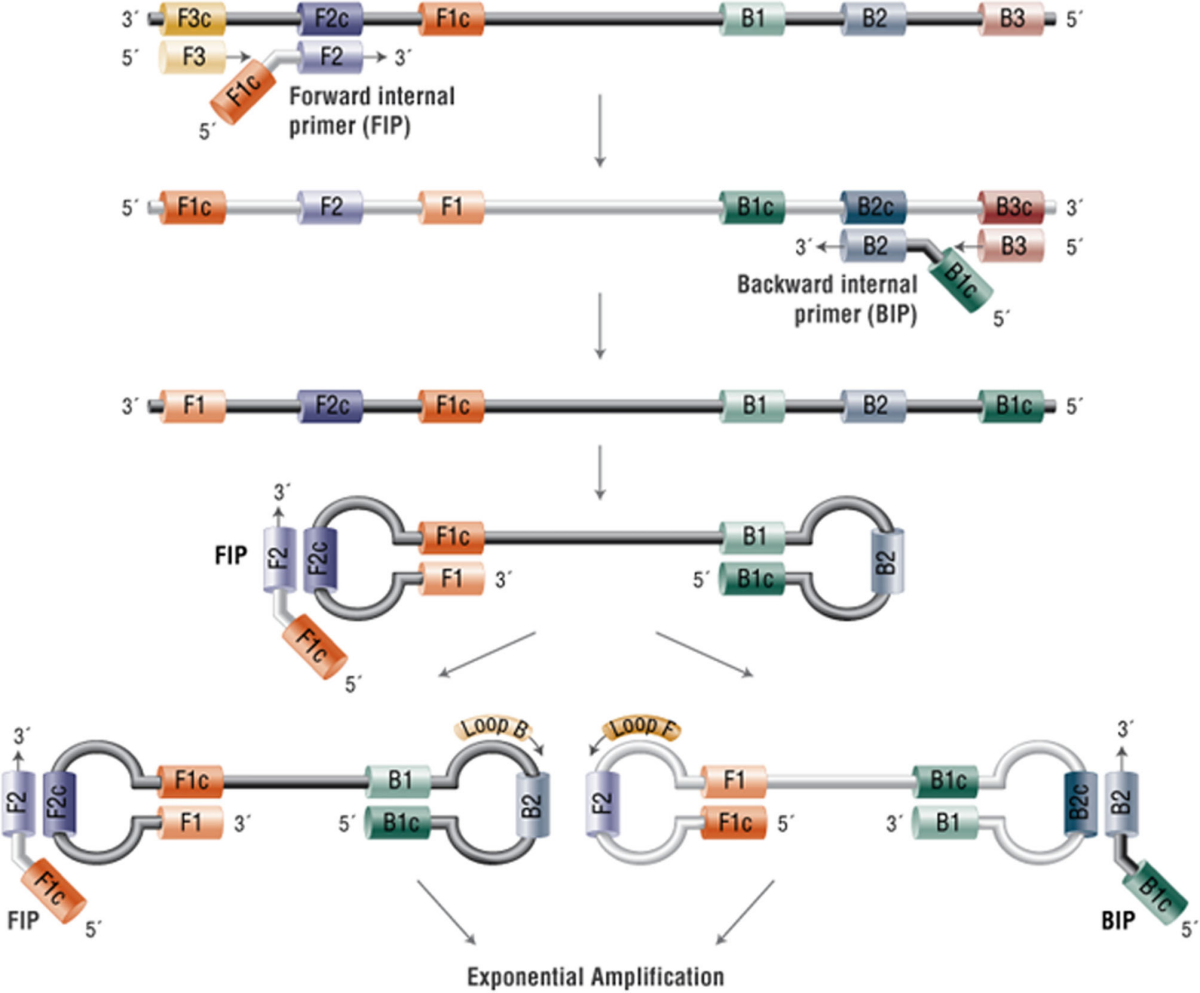
Mechanism of Loop-mediated isothermal amplification (LAMP). In the LAMP method, 4-6 primers are used to recognise 6-8 distinct regions of target DNA. A strand-displacing DNA polymerase initiates synthesis and 2 of the primers form loop structures to facilitate subsequent rounds of amplification. Adapted from New England Biolabs https://www.neb-online.de/en/pcr-and-dna-amplification/isothermal-amplification/.

A LAMP assay that is transferrable to a lab-on-a-chip was utilised using a conventional qPCR platform for readout. LAMP methods initially designed using 4 primers targeting 6 regions (16) and where the reaction proceeds without thermocycling is an ideal method for point-of-care testing. It relies on auto-cycling strand displacement DNA synthesis conducted by a DNA polymerase with high strand displacement activity. Subsequently, the LAMP method was extended to six primers targeting 8 regions (17) to accelerate the reaction. The six primers are Forward-Inner (FIP), Backward-Inner (BIP), Forward Outer (F3), Backward Outer (B3), Forward Loop (LF) and Backward Loop (LB). A stem-loop structure is constructed, in which the sequences of both DNA ends are derived from the inner primer. Subsequently, an exponential generation of inverted repeats is constructed as the inner primers anneal and cause amplification from the loops in the original structure (**Figure 1**). The addition of the loop primers LF and LB allow hybridisation of the available stem-loops that are not hybridised by the inner primers (FIP/BIP) and markedly accelerates the reaction from 1 hour to 10-15 minutes depending upon the concentration of the starting material. The primer sequences are given in **Table 1A**. The GenBank Accession numbers for the primers used are given in **Table 1B**.

**Table 1a:** pH-LAMP primer sequences.

Name	Sequence
F3_TERT	GCCTGAGCTGTACTTTGTCA
B3_TERT	GGTGAGCCACGAACTGTC
FIP_TERT	TGGGGTTTGATGATGCTGGCGA-GGGCGCGTACGACACCATCC
BIP_TERT	GGTCCAGAAGGCCGCCCAT-GCTGGAGGTCTGTCAAGGTA
LF_TERT	ACCTCCGTGAGCCTGTCCTG
LB_TERT	CACGTCCGCAAGGCCTTCA
F3_MYC	CCATGAGGAGACACCGCC
B3_MYC	TGCTGATGTGTGGAGACGT
FIP_MYC	AGCCTGCCTCTTTTCCACAGAA-CACCACCAGCAGCGAC
BIP_MYC	CTGGATCACCTTCTGCTGGAGG-GGCACCTCTTGAGGACCA
LF_MYC	TCATCTTCTTGTTCCTCCTCAGA
LB_MYC	CAGCAAACCTCCTCACAGCC
F3_TERC	TGTGAGCCGAGTCCTGG
B3_TERC	TCTCCGGAGGCACCCA
FIP_TERC	AGGAAGAGGAACGGAGCGAGTC-GTGCACGTCCCACAGCT
BIP_TERC	GAAAGGCCTGAACCTCGCCC-TGCCACCGCGAAGAGT
LB_TERC	AGAGACCCGCGGCTGACA
LF_TERC	CGGCGCGATTCCCTGA
F3_GAPRNA	GATGCTGGCGCTGAGTAC
B3_GAPRNA	GCTAAGCAGTTGGTGGTGC
FIP_GAPRNA	CTTTTGGCTCCCCCCTGCAAATGGAGTCCACTGGCGTCTT
BIP_GAPRNA	TCTGCTGATGCCCCCATGTTCGGAGGCATTGCTGATGATCT
LF_GAPRNA	AGCCTTCTCCATGGTGGTG
LB_GAPRNA	GTCATGGGTGTGAACCATGAG
F3_GAPDNA	ACCCCCATAGGCGAGATC
B3_GAPDNA	TGATGACCCTTTTGGCTCC
FIP_GAPDNA	CTCCATGGTGGTGAAGACGCC-CAAAATCAAGTGGGGCGATG
BIP_GAPDNA	CGGGAGGGGAAGCTGACTCA-ACAGCAGAGAAGCAGACAGT
LF_GAPDNA	TCCACGACGTACTCAGCG
LB_GAPDNA	GCAGGACCCGGGTTCAT

(FIP, forward inner primer; BIP, backward inner primer; LF, loop F; LB, loop B, **Figure 1**)

**Table 1b:** GenBank Accession numbers for primers used.

Primer	GenBank Accession number
**hTERT**	NG_009265.1, NM_198253.2, NM_001193376.1,NR_149162.1, R_149163.1
**TERC**	NG_016363.1
**MYC**	NG_007161.2, NM_002467.5, NM_001354870.1
**GAPDH**	NG_007073.2, NM_002046, NM_001256799,NM_001289745, NM_001289746 and NM_001357943
**HPV16**	K02718.1
**HPV18**	AY262282.1

Due to the small volume of DNA and RNA available following extraction of samples, only a single assay procedure was performed for each sample which required a final volume of 5 μL per reaction. Each mix obtained from a mastermix contained the following: 0.50 μL of 10 × isothermal pH-based buffer (pH 8.5–9), 0.30 μL of MgSO_4_ (100 mM stock), 0.28 μL of dNTPs (25 mM stock), 0.30 μL of BSA (20 mg/mL stock), 0.13 μL of NaOH (200 mM stock), 0.80 μL of Betaine (5M stock), 0.13 μL of Syto9 Green (20 μM stock), 0.02 μL of Bst 2.0 DNA polymerase (120,000 U/mL stock), 0.13 μL of avian myelobastosis virus (AMV, 25 U/μL stock, Promega), 0.05 μL of Rnase inhibitor (20 U/μL stock, ThermoFisher Scientific), 1 μL of extracted RNA or DNA and 0.50 μL of 10 × LAMP primer mixture (20 μM of BIP/FIP, 10 μM of LF/LB, and 2.5 μM B3/F3), and enough nuclease-free water (ThermoFisher Scientific) to bring the volume to 5 μL. In experiments targeting DNA, AMV and Rnase inhibitor were replaced by water in the reaction mix. This LAMP recipe has been previously published (18, 19). Reactions were performed at 63°C for 45 min. One melting cycle was performed at 0.1°C/s from 65°C up to 97°C for validation of the specificity of the products. Reactions were plated in 96-well plates and loaded into a LightCycler 96 real-time PCR system (LC96) (Roche Diagnostics). Following the LAMP assay a standard PCR assay was undertaken in duplicate for validation.

An hTERT mRNA result was considered positive if detection occurred within 30 min. Both the TERC DNA relative copy number and MYC mRNA expression relative to GAPDH DNA and mRNA respectively were calculated *via* the relative fold gene expression2^-(ddCt) method: a mean delta Ct (threshold cycle) was calculated from the patients in the control group, and used to calculate the relative fold change in the cancer patients. Any detection of HPV 16 or 18 DNA or RNA was considered positive. The results of the reference GenoID and Norchip tests were not available to the reader of the LAMP assay at the time of interpretation.

### Imaging

All scans were performed on a 3.0 Tesla Philips Achieva (Best, The Netherlands) with a dedicated in-house developed 37 mm ring-design solenoidal receiver coil that has been previously described (9, 32, 33). Cervical position was determined at vaginal examination, after which the coil was inserted and placed around the cervix. Image distortion from susceptibility artefacts were reduced by aspiration of vaginal air *via* a 4 mm diameter tube (Ryles; Pennine Healthcare, London, England).The intramuscular administration of Hyoscine butyl bromide (Buscopan) 20 mg decreased artefacts from bowel peristalsis.

T2-W images were obtained in three planes orthogonal to the cervix together with matched coronal Zonal Oblique Multislice (ZOOM) diffusion-weighted images (DWI). Sequence details are given in **Table 2**. ADC maps were automatically generated by the scanner software using a monoexponential fit of the data. These were compared with T2-W images to identify the presence and extent of a tumor within the cervix. Mass-lesions disrupting the normal cervical epithelial architecture that were intermediate signal-intensity on T2-W images with corresponding restriction on the ADC maps were recognized as tumor. Imaging reports were not available to the reader of the LAMP assay at the time of interpretation, nor was the radiologist aware of the results of the LAMP assay at the time of reporting.

**Table 2:** Scan parameters for endovaginal MRI.

Parameter	T2-W	ZOOM-DWI
TR (ms)	2500	6500
TE (ms)	80	54
FOV (mm x mm)	100 x 100	100 x 100
Slice thickness/gap (mm)	2.0/0.1	2.0/0.1
Voxel size (acquired) (mm^3^)	0.42 x 0.42 x 2.0	1.25 x 1.25 x 2.0
Voxel size (reconstructed) (mm^3^)	0.35 x 0.35 x 2.0	0.45 x 0.45 x 2.0
b-values (s/mm^2^)	N/A	0, 100, 300, 500, 800
No. slices	24 (coronal, axial); 22 (sagittal)	24 coronal
NSA	2	1

N/A, not applicable.

### Histopathology Analysis

Following definitive surgical excision, formalin fixed tissue specimens were sectioned at three to four millimeter intervals, embedded in paraffin and 2-3 micron sections mounted on glass slides. Hematoxylin and eosin (H&E) stained sections were reviewed by a specialist gynecological-oncology histopathologist and the presence or absence of residual tumor was recorded.

### Statistical Analysis

Statistical analysis used commercially-available software GraphPad Prism for Windows, (v8.3, GraphPad Software Inc., San Diego, CA, USA) and utilized primarily sensitivity and specificity analyses with 95% confidence limits (Wilson/Brown method) for comparison of the LAMP assay with the gold standard (qPCR or histology), the endovaginal MRI with histology and a combination of LAMP assay and endovaginal MRI with histology. Accuracy as defined by [(true positive) + (true negative)]/[(true positive) + (true negative) + (false positive) + (false negative)] were calculated. These analyses represent the clinical performance of the tests. As we did not perform repeat experiments due to low amount of starting material it was not possible to estimate the precision (degree to which the measurements were repeatable under the same conditions) of the experiments.

## Results

### Participants

Between August 2018 and June 2019, 27 patients with newly diagnosed Stage 1 cervical cancer (4 = 1a1, 9 = 1a2, 12 = 1b1, 2 = 1b2 by FIGO 2009 staging, Group 1) and 14 non-cancer HPV negative (normal) controls (Group 2) were prospectively recruited. Mean age and BMI were 34.7 years (range 25-51 years) and 23.7 (range 16.9-35.5) respectively. In Group 1, initial diagnosis was made with a LLETZ in 20 patients (where the tumor may have been removed in part or in entirety leaving no residual) and with punch biopsy in 7. Seventeen patients had squamous cell carcinoma (SCC), 10 had adenocarcinoma, with grade of disease distributed between well, moderate and poor differentiated in 5, 14 and 4 cases respectively (4 ungraded on histology). Lymphovascular space invasion was present in 7 cases, absent in 17 cases and not mentioned in 3. In Group 1, 26 of 27 patients had high-resolution MRI (I declined) and cervical swabs for LAMP assay analysis; patients in Group 2 had cervical swabs for LAMP assay analysis only.

**Figure 2:**
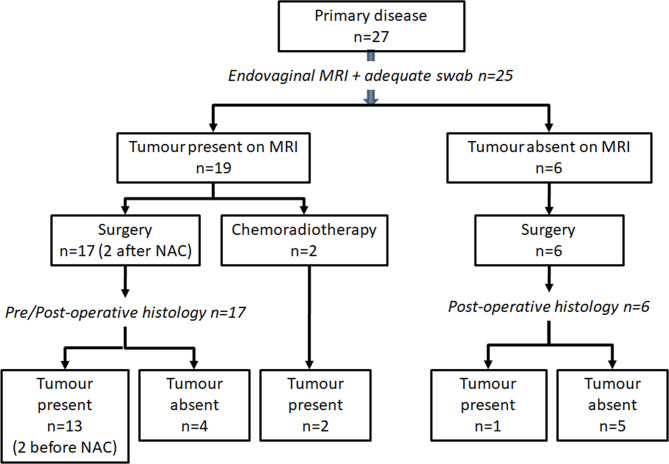
Patient cohort studied. Inclusion criteria and management in patients with newly diagnosed cervical cancer recruited to the MODULAR study.

In Group 1, 23 underwent primary surgery within 4 weeks of diagnosis (8 radical hysterectomy, 9 radical trachelectomy, 6 cold knife cone biopsy). On final histology, 12 of these patients had residual tumor. 2 further patients had radical trachelectomy after neoadjuvant chemotherapy and 2 had chemoradiotherapy following adverse findings at examination under anesthesia. These 4 patients were considered positive for tumor as swabs and diagnostic biopsies confirming tumor presence had been taken prior to treatment. Overall, 25 of 27 patients in Group 1 had endovaginal MRI, available histology and sufficient DNA/mRNA extracted on swabbing for inclusion in the analysis. DNA/mRNA extraction was sufficient for analysis in all patients from Group 2 (**Figure 2**).

### DNA/RNA Marker Yield

In Group 1, following DNA/RNA extraction the mean yield of DNA and RNA was 31.79ng/µL and 57.37ng/µL respectively. Nucleic acid purity (assessed by ratio of light absorbance at 260nm and 280nm) gave a mean of 1.62 for DNA (values >1.8 show high purity) and 2.01 for RNA (values >2 show high purity) indicating some contamination in the DNA samples, but that pure RNA was successfully extracted. The extracted nucleic acid yield was insufficient in 1 patient (negative on histology), so they were excluded from further analysis. In Group 2 mean yield of DNA and RNA was 43.28ng/µL and 56.07ng/µL respectively with a mean 260/280 ratio of 1.28 and 1.98 respectively, suggesting contaminants remained in the DNA samples but that RNA extraction was successful.

### Comparison of HPV-16 and HPV-18LAMP Assay With GenoID and Norchip Tests

In Group 1, 15 of 25 patients were positive for HPV 16 or 18 DNA and/or RNA on the gold standard DNA (GenoID) or E6/7 mRNA (Norchip) tests. Eleven were Type 16 and 4 were Type 18. Nine patients were negative for these HPV types, and in 1 case the results of the test were missing. Of these, 14 were positive on LAMP assay and 10 were negative, time to positive of clinical samples and the limits of detection for the synthetic HPV16 and 18 primers used are given in **Table 3**. All patients in Group 2 (14 HPV negative controls) were negative for HPV-16 and 18 DNA and RNA on the GenoID and Norchip tests. In this group, there were 2 false positives for the LAMP HPV-16 assay and 7 false positives with the LAMP HPV-18 DNA assay because of primer-dimer formation with the HPV-18 DNA LAMP primers in PCR negative cases. The LAMP HPV-18 mRNA assay was more reliable and detected 4 Type 18 mRNA detected by the Norchip test with 1 false positive. Overall sensitivities, specificities, positive and negative predictive values by HPV type are given in **Table 4**.

**Table 3:** Limits of detection and time to positive for synthetic sequences and clinical samples in Groups 1 and 2 of tumor markers and HPV 16 and 18.

Tumour marker	Limit of detection (copies/reaction)	Time to positive of synthetic sequence Minutes (Mean ± SD)	Time to positive of Group 1 (n = 25) Minutes (Mean ± SD)	Time to positive of Group 2 (n = 14) Minutes (Mean ± SD)
hTERT	10^3^	12.91 ± 0.44	37.4 ± 8.3 (in 13 positive cases)	42.9 ± 4.3 (in 7 positive cases)
MYC	10^1^	14.98 ± 1.95	18.3 ± 7.9 (in 23 positive cases)	18.0 ± 3.3 (in 11 positive cases)
GAPDH RNA	10^3^	9.35 ± 0.17	11.2 ± 1.0	11.2 ± 1.3
TERC DNA	10^1^	11.95 ± 0.15	15.0 ± 1.4 (in 23 positive cases)	15.8 ± 1.9 (in 10 positive cases)
GAPDH DNA	10^0^	13.62 ± 0.86	16.2 ± 2.6 (in 23 positive cases)	18.3 ± 4.6 (in 11 positive cases)
HPV 16 DNA	cf. primers as in Luo et al. (34)		18.0 ± 6.1 (in 11 positive cases)	21.8 (in 1 positive case)
HPV 16 mRNA	10^2^	15.27 ± 1.10	25.6 ± 7.2 (in 7 positive cases)	48.6 (in 1 positive case)
HPV 18 DNA	cf. primers as in Luo et al. (34)		28.8 ± 13.2 (in 16 positive cases)	42.9 ± 4.5 (in 7 positive cases)
HPV 18 mRNA	10^4^	17.06 ± 1.04	21.1 ± 4.6 (in 5 positive cases)	No positive cases

**Table 4:** Sensitivity, specificity, positive and negative predictive values of LAMP assays for detection of small volume Stage 1 cervical cancer alone and together with endovaginal MRI.

Assay for cancer detection	n	Reference standard	Sensitivity [%] (lower and upper 95%CI)	Specificity [%] (lower and upper 95%CI)	PPV [%] (lower and upper 95%CI)	NPV [%] (lower and upper 95%CI)
LAMP-HPV 16 DNA and/or mRNA	38	GenoID and Norchip test	90.9 (62.3, 99.5)	88.9 (71.9, 96.1)	76.9 (41.7, 91.8)	96.0 (80.5, 99.8)
LAMP-HPV 18 DNA and/or mRNA	38	GenoID and Norchip test	100.0 (80.6, 100)	22.2 (7.3, 38.5)	47.1 (31.5, 63.3)	100.0 (51.0, 100)
LAMP-hTERT	25	Histopathology	31.3 (14.2, 55.6)	77.8 (45.3, 96.1)	71.4 (35.9, 94.9)	38.9 (20.3, 61.4)
LAMP-TERC	24	Histopathology	40 (19.8, 64.3)	100 (70.1, 100)	100.0 (61.0, 100)	50.0 (29.0, 71.0)
LAMP-cMYC	25	Histopathology	43.8 (23.1, 66.8)	100 (70.1, 100)	100.0 (64.6, 100)	50.0 (29.0, 71.0)
LAMP-Scenario 1 (tumor marker positive regardless of HPV status	25	Histopathology	66.8 (44.4, 85.8)	77.8 (45.3, 96.1)	84.6 (57.8, 97.3)	77.8 (45.3, 96.1)
LAMP-Scenario 2 (tumor marker or HPV positive)	25	Histopathology	93.8 (71.7, 99.7)	55.8 (26.7, 81.1)	78.9 (56.7, 91.5)	83.3 (43.7, 99.1)
Endovaginal MRI	25	Histopathology	93.8 (71.7, 99.7)	44.4 (18.9, 73.3)	75.0 (53.1, 88.8)	80.0 (37.6, 99.0)
Endovaginal MRI+ Scenario 1	25	Histopathology	68.8 (44.4, 85.8)	88.9 (56.5, 99.4)	91.6 (64.6, 99.6)	61.5 (35.5, 82.3)
Endovaginal MRI+ Scenario 2	25	Histopathology	93.8 (71.7, 99.7)	77.8 (45.3, 96.1)	88.2 (65.7, 97.9)	87.5 (52.9, 99.4)

### Comparison of LAMP Tumor Marker Assay With Standard qPCR

**Figure 3:**
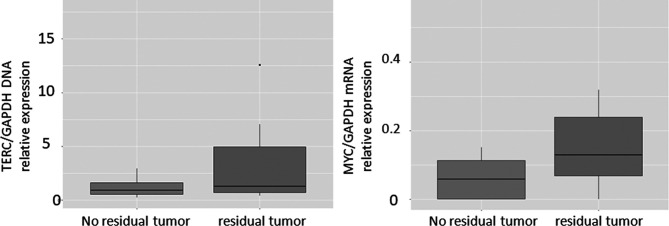
Boxplots of TERC/GAPDH and MYC/GAPDH in patients with cytology positive for cancer compared with negative controls.

In the non-cancer controls (Group 2), hTERT was positive at the outer limit of detection in 7 cases with a mean Ct of 42.9 minutes. Therefore, this was used as a Ct threshold for a positive result for the presence of cervical cancer. The positivity of TERC and MYC on LAMP assay was assessed by relative expression to GAPDH. Based on the 2^-(ddCt) for TERC/GAPDH and MYC/GAPDH from the controls in Group 2, the threshold of cancer detection for these markers was set at 3.12 and 0.155 respectively. Limits of detection and time to positive for the synthetic primers designed for hTERT, TERC, MYC, GAPDH and HPV mRNA and of clinical samples are given in **Table 3**. The relative expression of TERC DNA to GAPDH DNA and MYC mRNA to GAPDH mRNA in those without and with residual tumour in Group 1 is illustrated in **Figure 3**.

The hTERT LAMP assay results agreed with the PCR test for the presence or absence of cancer in 31 of 39 samples (sensitivity 57.1%, specificity 84.4%). The TERC/GAPDH DNA copy number PCR test was only successfully recorded in 26 of 39 samples; of these the standard deviation of the Ct for GAPDH was >0.25 in 14 cases and the standard deviation of the Ct for TERC was >0.25 in a further 4 cases, making the TERC/GAPDH replicable in only 8 cases. Similarly, with the MYC PCR assay a result was recorded in 25 of 39 samples; of these the standard deviation of the Ct for GAPDH was >0.25 in 10 cases and the standard deviation of the Ct for MYC was >0.25 in a further 10 cases, making the MYC/GAPDH replicable in only 5 cases. Comparison of LAMP assay with PCR was therefore not possible for TERC and MYC due to variability of the PCR results.

### Sensitivity and Specificity of Combined HPV and Tumor Marker LAMP Assay for Cancer Detection

Sensitivity and specificity for cancer detection was documented by comparison to the gold-standard of pathology. At the threshold set for hTERT, there was a sensitivity of 31.3% and specificity of 77.8%, accuracy 48% for tumor detection. Relative TERC/GAPDH DNA copy number was successfully recorded in 24 cases on LAMP assay and achieved a sensitivity of 40% and specificity of 100%, accuracy 62.5% at the threshold relative expression of 3.12. MYC/GAPDH relative expression was recorded in all 25 patients on LAMP assay and achieved a sensitivity of 43.8% and a specificity of 100%, accuracy 64% at a threshold relative expression of 0.155.

To evaluate the performance of the combined markers within the LAMP assay in detecting residual disease two scenarios were applied. In the first, tumor was considered present if any tumor marker (hTERT n=7; TERC/GAPDH>3.12 n=6, MYC/GAPDH>0.155 n=7) was present with or without HPV positivity and tumor absent if all tumor markers were absent regardless of the presence or not of HPV. Using these criteria, gave the LAMP a sensitivity of 68.8% (5 false negatives), specificity of 77.8% (2 false positives), positive predictive value of 84.6% and a negative predictive value of 77.8%, accuracy 72%. In the second scenario, tumor was considered present if any tumor marker or HPV was present, and tumor was considered absent if all tumor markers and HPV were absent. Using these criteria, the LAMP had a sensitivity of 93.8% (1 false negative), specificity of 55.8% (4 false positives), positive predictive value of 78.9% and a negative predictive value of 83.3%, accuracy 80% (**Table 3**).

### Sensitivity and Specificity of High-Resolution MRI

**Figure 4:**
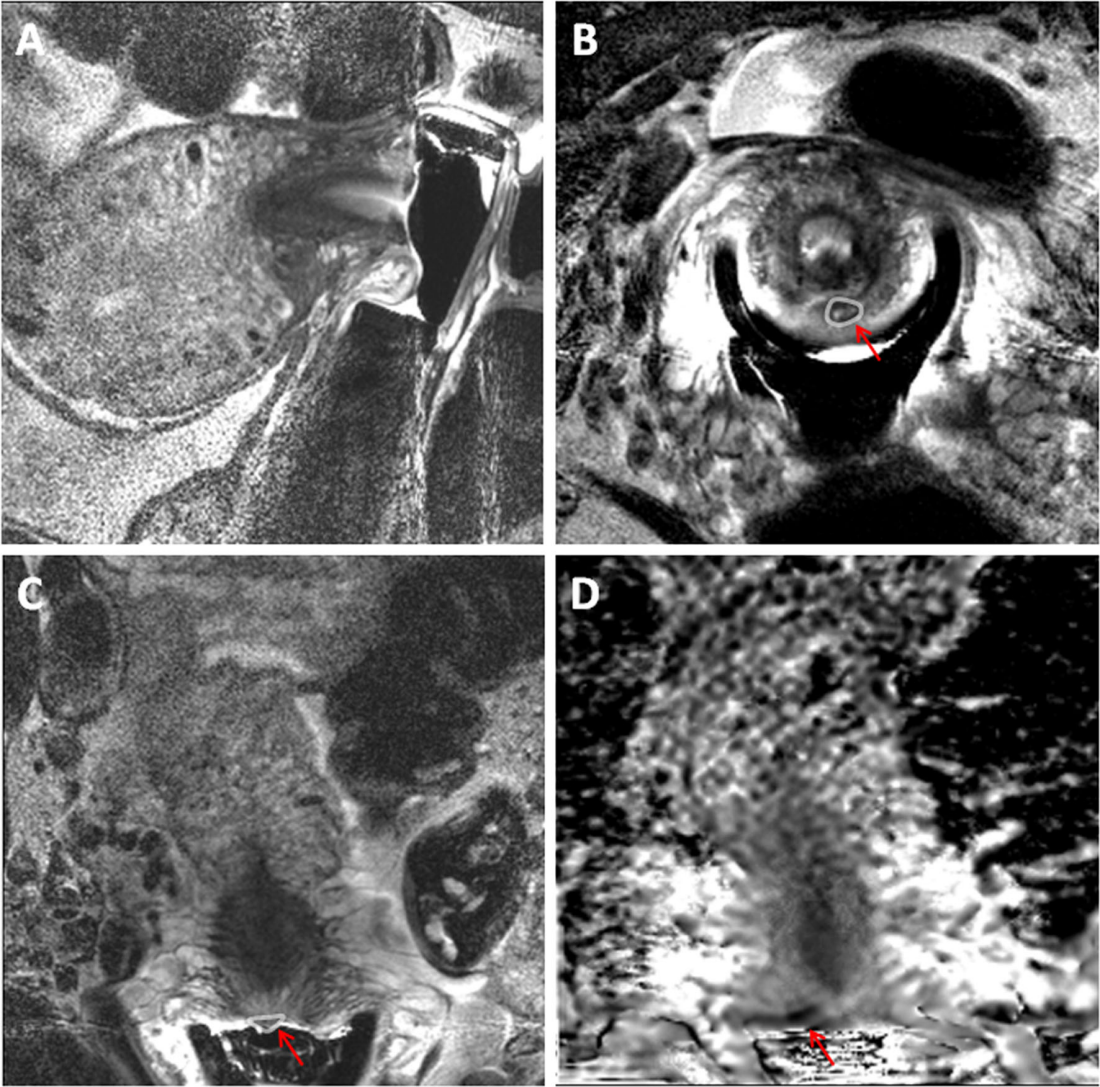
32-year old female with an endovaginal MRI that was a false positive for cervical cancer. T2-weighted sagittal **(A)**, axial **(B)** and coronal **(C)** MRI scans obtained using an endovaginal coil with corresponding ADC map in the coronal plane **(D)**. A small nodule on the posterior ectocervix in C (arrow) with focal diffusion restriction in D was considered positive for residual tumour. On LAMP assay from a cervical cytology swab, the cells were negative for all tumour markers and for HPV E6/7 mRNA indicating that the MRI result was likely a false positive. This was confirmed at histology from a subsequent repeat cone biopsy.

Of the 25 patients scanned, 20 had tumor present on MRI and 15 of these were confirmed at histology (11 at surgery, 4 on biopsy prior to chemoradiotherapy). In the 5 patients who were negative for tumor on MRI, 4 cases had no residual disease on histology and 1 was positive for tumor (**Figure 4**). Sensitivity and specificity of MRI was therefore 93.8% and 44.4% respectively, accuracy 76%, PPV 75.0%, NPV 80.0%.

### Value of LAMP Assay Testing Combined With High Resolution MRI

If patients in Group 1 were considered positive only if they were positive on MRI and LAMP tumor markers (scenario 1), sensitivity was 68.8% but specificity improved to 88.9%, accuracy 76%; for scenario 2 sensitivity was 93.8%specificity 87.5% and accuracy 88% (**Table 4**).

## Discussion

This data indicates the feasibility of performing HPV and tumor marker testing using LAMP technology and indicates the assays current accuracy in comparison to standard PCR systems. The ability to perform multiple marker testing at point-of-care indicates the potential added value of this type of molecular testing in the diagnostic pathway of patients with early stage, small volume cervical cancers following cone biopsy or LLETZ, who undergo MRI for determining the presence and extent of residual disease prior to definitive surgery. The increase in accuracy is critical because the evidence for increased obstetric risk following CKC or LLETZ is substantial: although no difference in first-trimester miscarriage rates was reported in a meta-analysis (35) and subsequent Cochrane review (36), a population based study suggested an almost four-fold increase in the risk of mid-trimester loss in women post-conization (n=15 108) compared to untreated individuals (n=2 164 006; 1.5% *versus* 0.4%; RR 4.0 and 95% CI 3.3–4.8) (37). A metanalysis of 20 studies showed that the frequency and severity of these complications increased with methods that are known to remove large amounts of cervical tissue (38) and was confirmed by a later meta-analysis (39) and a Cochrane review (40). The combination of MRI and HPV and tumour marker testing thus enables decision making for optimal surgical approach in patients wishing to preserve fertility. The increased accuracy of the LAMP assay also comes with major advantages: the time to positive (TTP) of less than 25 minutes for all tests demonstrates the true point-of-care potential of this assay to deliver rapid, accurate results when utilised on a portable lab-on-a-chip platform.

Validation of the methodology against conventional PCR showed largely equivalent results for both the DNA and RNA tests in the HPV and hTERT primers. The PCR primers for MYC and TERC/GAPDH performed poorly in the experiments, perhaps indicating alternative PCR primers should be used as their technical sensitivity was below expectation in the clinical samples. Similarly, validation of the HPV-16 and the tumor markers against standard PCR was also equivalent. The pH-LAMP HPV Type 18 DNA marker, as developed by Luo et al. (34), did not perform as expected and resulted in a large number of false positives through unexpected primer-dimer formation in clinical samples. This could be ameliorated by setting a very short time to positive threshold but alternative HPV 18 DNA LAMP primer sets would be available to test which may provide more reliable real-world results. The development of a robust, sensitive set of HPV DNA type specific pH-LAMP primers would be a prerequisite prior to the platform being successful as a screening platform.

Approaches combining HPV DNA testing with cytology have been previously tried (41) to optimise the sensitivity and specificity of cancer detection at the time of colposcopy in patients referred because of abnormal smears. HPV DNA testing is very sensitive (~95%) but lacks the specificity (30-50%) required for cervical cancer detection (42). There are a wide range of commercially available HPV detection assays which are based on different techniques such as target amplification (mainly PCR), signal amplification, and probe amplification (43). Food and Drug Administration (FDA) approved assays for HPV DNA are aimed at a panel of 13 or14 high-risk HPVs. Nevertheless, none of these is used routinely in a screening setting, and their low specificity makes them unsuitable for screening. HPV E6/E7 mRNA tests (as described here) have superior specificity to HPV DNA tests (42, 43). Overexpression of HPV E6 and E7 mRNAs has been evaluated as a marker for the transition from a productive infection to an abortive infection that eventually promotes cell transformation. Thus, the advantage of our spatially multiplexed LAMP assay system is that it also allows utilization of HPV mRNA which can substantially improve the specificity for cancer detection in patients at high risk of invasive disease.

The three pH-LAMP tests (hTERT mRNA, MYC mRNA and TERC DNA) each had poor sensitivity but excellent specificity for predicting the presence of residual tumor. The need for three pH-LAMP nucleic tests is warranted to cover a range of possible scenarios; comparison between these showed that all 3 were positive in 1 case, 2 were positive in 6 cases and 1 was positive in 7 cases. The poor sensitivity of the tumor marker tests is counteracted by having the HPV markers included as part of the assay, as their sensitivity for detecting cancer was high. Unfortunately, the HPV 18 DNA LAMP assay designed by Luo et al. (34) in synthetic sequence testing did not reveal false positive tests but was unreliable in our clinical samples as false positive results were in abundance due to primer-dimer formation. However, the newly designed HPV 18 mRNA assay included evaluating primer-dimer formation using NuPack assessment and was highly sensitive and specific. Our analysis however, considered a sample to be HPV positive if either DNA or RNA was positive, so that the HPV DNA data reduced the overall specificity of the result. Jointly utilising HPV and tumor marker testing and interpreting the tumor marker status on those with positive HPV results would help differentiate the true positives from those with an indeterminate result that require further investigation. Conversely, it is also true that the few false negative cases seen with HPV DNA testing may be successfully detected by a positive tumor marker status.

Other markers could be considered for inclusion on spatially multiplexed chip technology in future. Because the expression of HPV viral E7 leads to an increase of cyclin-dependent kinase inhibitor p16 (p16INK4a), p16 would be a possible candidate. However, as p16 overexpression, fundamentally is a marker of HPV infection, it was not selected for the current study. It provides a similar sensitivity and specificity profile to the HPV markers. A meta-analysis of seventeen studies showed a pooled sensitivity and specificity of p16INK4a to detect CIN2 or worse in patients with squamous intraepithelial lesions was 83.8% and 65.7% respectively (44). DNA methylation of several human genes has been shown to be also a relevant event for cervical carcinoma development. The use of differential methylation hybridization using a pilot methylation array allowed the identification of SOX1, NKX6-1, PAX1, WT1, and LMX1A as frequently methylated genes in cervical cancer and precursor lesions (45). In future, optimal marker selection and methods to identify DNA methylation may substantially improve the sensitivity of the tumor marker testing.

Nucleic acid based tests have yet to be evaluated at a population screening level: the change to HPV DNA primary screening has only recently been adopted (46), especially in the UK. The introduction of the HPV vaccine has reduced the number of CIN2/3 diagnoses in Scotland (47), so the economic benefit of testing for HPV within a screening programme remains debatable, especially where high quality cytology services are available. In areas with limited cervical screening programmes and without the high quality, well-resourced colposcopic service seen in developed countries, however, the benefit of a rapid, low-cost, point-of-care approach to cervical screening could potentially be transformational. It would provide the opportunity for developing countries to skip over several hurdles which developed countries have encountered in establishing their screening programmes.

LOC technology is versatile to a wide range of targets including bacterial and viral transcripts (20) and sample types when coupled with a sample preparation module. Additionally, its use of standard electronic components promotes scalability and portability which ideally match the requirements of portable diagnostics and allow for future pathogen multiplexing capabilities. Other reported commercial isothermal assays for COVID-19 detection such as Lucira’s COVID-19 All-In-One Test Kit is a good example of a molecular *in vitro* diagnostic test that generates results in 30 minutes with analytical sensitivity comparable to the RT-PCR assays. However, it is limited to COVID-19 hence does not allow multi-pathogen detection, and the sensitivity is expected to be reduced due to the all-in-one test kit approach when compared to the full sample extraction methodology. We optimised our isothermal methods to enable the compatibility to our microchip technology as an alternative to fluorescence and time-consuming incubation. This approach has been shown to hold significant potential for the development of a cheap, portable and quantitative diagnostic tool (48) using an external thermal controller in conjunction with a desktop computer. Moreover, or recent work has demonstrated a fully portable LOC platform which has integrated thermal management within the diagnostic platform and uses a smartphone application (Android OS) for data acquisition, visualization and cloud connectivity and has been used to detect breast cancer mutations (49), genes related to antimicrobial resistance (18) and COVID-19 (19).

There are several limitations to our study. Firstly, we did not include any HPV positive non-cancer controls in this study. This would be the ideal if testing HPV alone as a biomarker for the presence of tumor, however, as the aim was to develop a spatially multiplexed assay with HPV and tumor markers, we felt that a control group that was negative for cancer and HPV would be more definitive in the first instance in this pilot study. Secondly, validation of the LAMP tumor marker assay was limited by the variability of the standard PCR results. This was partly because of the limited volume of extracted RNA available from these cytology samples for the multiple LAMP and PCR assays; uncertainty around the concentrations of RNA and DNA available from the cytology swabs also meant that we may have used a larger sample volume than necessary for each LAMP experiment and compromised the number of successful repeat experiments, particularly for the PCR validation. Moreover, the purity of the DNA samples was low so that contaminants and inhibitors within biological samples may have affected the performance of the PCR assay (50) This is likely to have been more pronounced from cytology samples where cellular content is low. Nevertheless, a key benefit of the LAMP method is robust detection even in crude samples (51) which lends itself to point-of-care testing possibly even on direct cervical brush samples. Developing a new methodology to better extract DNA/RNA from the tested samples would be of value but was outside the scope of this work. Other intrinsic limitations were lack of repeated testing due to insufficient starting materials which prevented us estimating the precision of our results. Therefore, reproducibility of the LAMP assay for cervical cancer biopsies remains to be established. Reduction of the cellular material for the PCR validation also may well have reduced the repeatability of the tumor marker PCR assay (52) and prevented validation of our LAMP assay for TERC/GAPDH and MYC GAPDH. The experiments will also need to be repeated on a larger sample size. Nevertheless, translation of a LAMP assay technique for spatially multiplexed tumor markers and HPV to a lab-on-a-chip is achievable, but the low sensitivity of the tumor markers and low specificity of the HPV markers mean that these markers are best tested for together to be clinically useful. It will require integration of sample preparation and nucleic acid extraction with the LAMP assay to achieve a deliverable test at point-of-care.

In summary, this work has demonstrated the feasibility of a LAMP assay comprising HPV 16 and 18 DNA/RNA and tumor markers hTERT, TERC and MYC for early detection of cervical cancer using prospectively collected cytology samples from patients with newly diagnosed cervical cancer and in normal controls. While the specificity for cancer detection was superior with the tumor markers, sensitivity was relatively low; the reverse was true for HPV detection. In patients with small cervical tumors suitable for fertility-sparing surgery, use of a spatially multiplexed LAMP assay in conjunction with high resolution endovaginal resulted in improved specificity for cancer detection.

## Data Availability Statement

The imaging data from this study are available via the Institute of Cancer Research’s XNAT imaging data repository. Access requests will be granted depending on appropriate regulatory and institutional approvals upon contacting the corresponding author.

## Ethics Statement

The studies involving human participants were reviewed and approved by London-Surrey Research Ethics Committee Ref: REC18/LO/0865. The patients/participants provided their written informed consent to participate in this study.

## Author Contributions

NdS, TI, and PG devised the study. BW, NM, IP, JR-M, and NdS contributed to the data acquisition/collection. KV and AA performed the histopathological analysis. BW, IP, JR-M, and NdS analyzed the data. All authors contributed to the article and approved the submitted version.

## Funding

We acknowledge Cancer Research UK Centre of Excellence funding to Imperial College and The Institute of Cancer Research (C309/A26234). Cancer Research UK and the Engineering and Physical Sciences Research Council also supported the Cancer Imaging Centre at the Institute of Cancer Research and Royal Marsden Hospital in association with the Medical Research Council and Department of Health C1060/A10334, C1060/A16464. We also acknowledge National Institute for Health Research funding to the Biomedical Research Centre at Royal Marsden Hospital/Institute of Cancer Research and to the Clinical Research Facility in Imaging.

## Author Disclaimer

The views expressed in this publication are those of the author(s) and not necessarily those of the National Health Service, the National Institute for Health Research or the Department of Health.

## Conflict of Interest

The authors declare that the research was conducted in the absence of any commercial or financial relationships that could be construed as a potential conflict of interest.

## Publisher’s Note

All claims expressed in this article are solely those of the authors and do not necessarily represent those of their affiliated organizations, or those of the publisher, the editors and the reviewers. Any product that may be evaluated in this article, or claim that may be made by its manufacturer, is not guaranteed or endorsed by the publisher.

## References

[1] Lindsay R Paul J Siddiqui N Davis J Gaffney DK. Survey on the Management of Early Cervical Cancer Among Members of the GCIG. Int J Gynecol Cancer (2012) 22(9):1617–23. doi: 10.1097/IGC.0b013e31826fd66b 23038419

[2] Martin-Hirsch PP Paraskevaidis E Bryant A Dickinson HO. Surgery for Cervical Intraepithelial Neoplasia. Cochrane Database Syst Rev (2013) 2013(12):CD001318. doi: 10.1002/14651858.CD001318.pub3 PMC8958508 24302546

[3] Kyrgiou M Bowden SJ Athanasiou A Paraskevaidi M Kechagias K Zikopoulos A. Morbidity After Local Excision of the Transformation Zone for Cervical Intra-Epithelial Neoplasia and Early Cervical Cancer. Best Pract Res Clin Obstet Gynaecol (2021) 75:10–22. doi: 10.1016/j.bpobgyn.2021.05.007 34148778

[4] Noehr B Jensen A Frederiksen K Tabor A Kjaer SK. Depth of Cervical Cone Removed by Loop Electrosurgical Excision Procedure and Subsequent Risk of Spontaneous Preterm Delivery. Obstet Gynecol (2009) 114(6):1232–8. doi: 10.1097/AOG.0b013e3181bf1ef2 19935024

[5] Castanon A Landy R Brocklehurst P Evans H Peebles D Singh N. Risk of Preterm Delivery With Increasing Depth of Excision for Cervical Intraepithelial Neoplasia in England: Nested Case-Control Study. BMJ (2014) 349:g6223. doi: 10.11361bmj.e5174 25378384 10.1136/bmj.g6223 PMC4220819

[6] Pils S Eppel W Seemann R Natter C Ott J. Sequential Cervical Length Screening in Pregnancies After Loop Excision of the Transformation Zone Conisation: A Retrospective Analysis. BJOG (2014) 121(4):457–62. doi: 10.1111/1471-0528.12390 24148580

[7] Kasuga Y Miyakoshi K Nishio H Akiba Y Otani T Fukutake M. Mid-Trimester Residual Cervical Length and the Risk of Preterm Birth in Pregnancies After Abdominal Radical Trachelectomy: A Retrospective Analysis. BJOG (2017) 124(11):1729–35. doi: 10.1111/1471-0528.14688 28418597

[8] Alvarez RM Biliatis I Rockall A Papadakou E Sohaib SA deSouza NM. MRI Measurement of Residual Cervical Length After Radical Trachelectomy for Cervical Cancer and the Risk of Adverse Pregnancy Outcomes: A Blinded Imaging Analysis. BJOG (2018) 125(13):1726–33. doi: 10.1111/1471-0528.15429 30099822

[9] deSouza NM Dina R McIndoe GA Soutter WP. Cervical Cancer: Value of an Endovaginal Coil Magnetic Resonance Imaging Technique in Detecting Small Volume Disease and Assessing Parametrial Extension. Gynecol Oncol (2006) 102(1):80–5. doi: 10.1016/j.ygyno.2005.11.038 16427688

[10] Downey K Jafar M Attygalle AD Hazell S Morgan VA Giles SL. Influencing Surgical Management in Patients With Carcinoma of the Cervix Using a T2- and ZOOM-Diffusion-Weighted Endovaginal MRI Technique. Br J Cancer (2013) 109(3):615–22. doi: 10.1038/bjc.2013.375 PMC3738120 23868012

[11] de Sanjose S Quint WG Alemany L Geraets DT Klaustermeier JE Lloveras B. Human Papillomavirus Genotype Attribution in Invasive Cervical Cancer: A Retrospective Cross-Sectional Worldwide Study. Lancet Oncol (2010) 11(11):1048–56. doi: 10.1016/S1470-2045(10)70230-8 20952254

[12] Bodelon C Vinokurova S Sampson JN den Boon JA Walker JL Horswill MA. Chromosomal Copy Number Alterations and HPV Integration in Cervical Precancer and Invasive Cancer. Carcinogenesis (2016) 37(2):188–96. doi: 10.1093/carcin/bgv171 PMC4834967 26660085

[13] Oyervides-Muñoz MA Pérez-Maya AA Rodríguez-Gutiérrez HF Gómez-Macias GS Fajardo-Ramírez OR Treviño V. Understanding the HPV Integration and Its Progression to Cervical Cancer. Infect Genet Evol (2018) 61:134–44. doi: 10.1016/j.meegid.2018.03.003 29518579

[14] Tjalma WA Depuydt CE. Cervical Cancer Screening: Which HPV Test Should be Used–L1 or E6/E7? Eur J Obstet Gynecol Reprod Biol (2013) 170(1):45–6. doi: 10.1016/j.ejogrb.2013.06.027 23932300

[15] Koliopoulos G Nyaga VN Santesso N Bryant A Martin-Hirsch PP Mustafa RA. Cytology Versus HPV Testing for Cervical Cancer Screening in the General Population. Cochrane Database Syst Rev (2017) 8:CD008587. doi: 10.1002/14651858.CD008587.pub2 28796882 PMC6483676

[16] Notomi T Okayama H Masubuchi H Yonekawa T Watanabe K Amino N. Loop-Mediated Isothermal Amplification of DNA. Nucleic Acids Res (2000) 28(12):E63. doi: 10.1093/nar/28.12.e63 10871386 PMC102748

[17] Nagamine K Hase T Notomi T. Accelerated Reaction by Loop-Mediated Isothermal Amplification Using Loop Primers. Mol Cell Probes (2002) 16(3):223–9. doi: 10.1006/mcpr.2002.0415 12144774

[18] Rodriguez-Manzano J Moser N Malpartida-Cardenas K Moniri A Fisarova L Pennisi I. Rapid Detection of Mobilized Colistin Resistance Using a Nucleic Acid Based Lab-On-a-Chip Diagnostic System. Sci Rep (2020) 10(1):8448. doi: 10.1038/s41598-020-64612-1 32439986 PMC7242339

[19] Rodriguez-Manzano J Malpartida-Cardenas K Moser N Pennisi I Cavuto M Miglietta L. Handheld Point-Of-Care System for Rapid Detection of SARS-CoV-2 Extracted RNA in Under 20 Min. ACS Cent Sci (2021) 7(2):307–17. doi: 10.1021/acscentsci.0c01288 PMC7839415 33649735

[20] Pennisi I Rodriguez-Manzano J Moniri A Kaforou M Herberg JA Levin M. Translation of a Host Blood RNA Signature Distinguishing Bacterial From Viral Infection Into a Platform Suitable for Development as a Point-Of-Care Test. JAMA Pediatr (2021) 175(4):417–9. doi: 10.1001/jamapediatrics.2020.5227 PMC7783591 33393977

[21] Hagiwara M Sasaki H Matsuo K Honda M Kawase M Nakagawa H. Loop-Mediated Isothermal Amplification Method for Detection of Human Papillomavirus Type 6, 11, 16, and 18. J Med Virol (2007) 79(5):605–15. doi: 10.1002/jmv.20858 PMC7166959 17385684

[22] Satoh T Matsumoto K Fujii T Sato O Gemma N Onuki M. Rapid Genotyping of Carcinogenic Human Papillomavirus by Loop-Mediated Isothermal Amplification Using a New Automated DNA Test (Clinichip HPV). J Virol Methods (2013) 188(1-2):83–93. doi: 10.1016/j.jviromet.2012.10.014 23219807

[23] Mutirangura A Sriuranpong V Termrunggraunglert W Tresukosol D Lertsaguansinchai P Voravud N. Telomerase Activity and Human Papillomavirus in Malignant, Premalignant and Benign Cervical Lesions. Br J Cancer (1998) 78(7):933–9. doi: 10.1038/bjc.1998.604 PMC2063133 9764586

[24] Snijders PJ van Duin M Walboomers JM Steenbergen RD Risse EK Helmerhorst TJ. Telomerase Activity Exclusively in Cervical Carcinomas and a Subset of Cervical Intraepithelial Neoplasia Grade III Lesions: Strong Association With Elevated Messenger RNA Levels of Its Catalytic Subunit and High-Risk Human Papillomavirus DNA. Cancer Res (1998) 58(17):3812–8. 9731489

[25] Wang HY Park S Kim S Lee D Kim G Kim Y. Use of hTERT and HPV E6/E7 mRNA RT-qPCR TaqMan Assays in Combination for Diagnosing High-Grade Cervical Lesions and Malignant Tumors. Am J Clin Pathol (2015) 143(3):344–51. doi: 10.1309/AJCPF2XGZ2XIQYQX 25696792

[26] Wang X Liu J Xi H Cai L. The Significant Diagnostic Value of Human Telomerase RNA Component (hTERC) Gene Detection in High-Grade Cervical Lesions and Invasive Cancer. Tumour Biol (2014) 35(7):6893–900. doi: 10.1007/s13277-014-1915-z 24737583

[27] Kubler K Heinenberg S Rudlowski C Keyver-Paik MD Abramian A Merkelbach-Bruse S. C-Myc Copy Number Gain Is a Powerful Prognosticator of Disease Outcome in Cervical Dysplasia. Oncotarget (2015) 6(2):825–35. doi: 10.18632/oncotarget.2706 PMC4359258 25596731

[28] Kuglik P Kasikova K Smetana J Vallova V Lastuvkova A Moukova L. Molecular Cytogenetic Analyses of hTERC (3q26) and MYC (8q24) Genes Amplifications in Correlation With Oncogenic Human Papillomavirus Infection in Czech Patients With Cervical Intraepithelial Neoplasia and Cervical Carcinomas. Neoplasma (2015) 62(1):130–9. doi: 10.4149/neo_2015_017 25563377

[29] Hammer A Rositch A Qeadan F Gravitt PE Blaakaer J. Age-Specific Prevalence of HPV16/18 Genotypes in Cervical Cancer: A Systematic Review and Meta-Analysis. Int J Cancer (2016) 138(12):2795–803. doi: 10.1002/ijc.29959 26661889

[30] Jeney C Takacs T Sebe A Schaff Z. Detection and Typing of 46 Genital Human Papillomaviruses by the L1F/L1R Primer System Based Multiplex PCR and Hybridization. J Virol Methods (2007) 140(1-2):32–42. doi: 10.1016/j.jviromet.2006.10.013 17169438

[31] Kraus I Molden T Holm R Lie AK Karlsen F Kristensen GB. Presence of E6 and E7 mRNA From Human Papillomavirus Types 16, 18, 31, 33, and 45 in the Majority of Cervical Carcinomas. J Clin Microbiol (2006) 44(4):1310–7. doi: 10.1128/JCM.44.4.1310-1317.2006 PMC1448674 16597856

[32] Gilderdale DJ deSouza NM Coutts GA Chui MK Larkman DJ Williams AD. Design and Use of Internal Receiver Coils for Magnetic Resonance Imaging. Br J Radiol (1999) 72(864):1141–51. doi: 10.1259/bjr.72.864.10703469 10703469

[33] Charles-Edwards E Morgan V Attygalle AD Giles SL Ind TE Davis M. Endovaginal Magnetic Resonance Imaging of Stage 1A/1B Cervical Cancer With A T2- and Diffusion-Weighted Magnetic Resonance Technique: Effect of Lesion Size and Previous Cone Biopsy on Tumor Detectability. Gynecol Oncol (2011) 120(3):368–73. doi: 10.1016/j.ygyno.2010.10.013 21093895

[34] Luo L Nie K Yang MJ Wang M Li J Zhang C. Visual Detection of High-Risk Human Papillomavirus Genotypes 16, 18, 45, 52, and 58 by Loop-Mediated Isothermal Amplification With Hydroxynaphthol Blue Dye. J Clin Microbiol (2011) 49(10):3545–50. doi: 10.1128/JCM.00930-11 PMC3187293 21865423

[35] Kyrgiou M Mitra A Arbyn M Stasinou SM Martin-Hirsch P Bennett P. Fertility and Early Pregnancy Outcomes After Treatment for Cervical Intraepithelial Neoplasia: Systematic Review and Meta-Analysis. BMJ (2014) 349:g6192. doi: 10.1136/bmj.g6192 25352501 PMC4212006

[36] Kyrgiou M Mitra A Arbyn M Paraskevaidi M Athanasiou A Martin-Hirsch PP. Fertility and Early Pregnancy Outcomes After Conservative Treatment for Cervical Intraepithelial Neoplasia. Cochrane Database Syst Rev (2015) 9):CD008478. doi: 10.1002/14651858.CD008478.pub2 PMC6457639 26417855

[37] Albrechtsen S Rasmussen S Thoresen S Irgens LM Iversen OE. Pregnancy Outcome in Women Before and After Cervical Conisation: Population Based Cohort Study. BMJ (2008) 337:a1343. doi: 10.1136/bmj.a1343 18801869 PMC2544429

[38] Arbyn M Kyrgiou M Simoens C Raifu AO Koliopoulos G Martin-Hirsch P. Perinatal Mortality and Other Severe Adverse Pregnancy Outcomes Associated With Treatment of Cervical Intraepithelial Neoplasia: Meta-Analysis. BMJ (2008) 337:a1284. doi: 10.1136/bmj.a1284 18801868 PMC2544379

[39] Kyrgiou M Athanasiou A Paraskevaidi M Mitra A Kalliala I Martin-Hirsch P. Adverse Obstetric Outcomes After Local Treatment for Cervical Preinvasive and Early Invasive Disease According to Cone Depth: Systematic Review and Meta-Analysis. BMJ (2016) 354:i3633. doi: 10.1097/01.ogx.0000508341.95858.c5 27469988 PMC4964801

[40] Kyrgiou M Athanasiou A Kalliala IEJ Paraskevaidi M Mitra A Martin-Hirsch PP. Obstetric Outcomes After Conservative Treatment for Cervical Intraepithelial Lesions and Early Invasive Disease. Cochrane Database Syst Rev (2017) 11:CD012847. doi: 10.1002/14651858.CD012847 29095502 PMC6486192

[41] Tornesello ML Buonaguro L Giorgi-Rossi P Buonaguro FM. Viral and Cellular Biomarkers in the Diagnosis of Cervical Intraepithelial Neoplasia and Cancer. BioMed Res Int (2013) 2013:519619. doi: 10.1155/2013/519619 24383054 PMC3872027

[42] Arbyn M Ronco G Anttila A Meijer CJ Poljak M Ogilvie G. Evidence Regarding Human Papillomavirus Testing in Secondary Prevention of Cervical Cancer. Vaccine (2012) 30(Suppl 5):F88–99. doi: 10.1016/j.vaccine.2012.06.095 23199969

[43] Luhn P Wentzensen N. HPV-Based Tests for Cervical Cancer Screening and Management of Cervical Disease. Curr Obstet Gynecol Rep (2013) 2(2):76–85. doi: 10.1007/s13669-013-0040-0 23705102 PMC3658152

[44] Roelens J Reuschenbach M von Knebel Doeberitz M Wentzensen N Bergeron C Arbyn M. P16ink4a Immunocytochemistry Versus Human Papillomavirus Testing for Triage of Women With Minor Cytologic Abnormalities: A Systematic Review and Meta-Analysis. Cancer Cytopathol (2012) 120(5):294–307. doi: 10.1002/cncy.21205 22700382 PMC4198379

[45] Lai HC Lin YW Huang TH Yan P Huang RL Wang HC. Identification of Novel DNA Methylation Markers in Cervical Cancer. Int J Cancer (2008) 123(1):161–7. doi: 10.1002/ijc.23519 18398837

[46] Rebolj M Rimmer J Denton K Tidy J Mathews C Ellis K. Primary Cervical Screening With High Risk Human Papillomavirus Testing: Observational Study. BMJ (Clinical Res ed) (2019) 364:l240–l. doi: 10.1136/bmj.l240 PMC6364146 30728133

[47] Palmer T Wallace L Pollock KG Cuschieri K Robertson C Kavanagh K. Prevalence of Cervical Disease at Age 20 After Immunisation With Bivalent HPV Vaccine at Age 12-13 in Scotland: Retrospective Population Study. BMJ (Clinical Res ed) (2019) 365:l1161. doi: 10.1136/bmj.l1161 PMC6446188 30944092

[48] Malpartida-Cardenas K Miscourides N Rodriguez-Manzano J Yu LS Moser N Baum J. Quantitative and Rapid Plasmodium Falciparum Malaria Diagnosis and Artemisinin-Resistance Detection Using a CMOS Lab-On-Chip Platform. Biosens Bioelectron (2019) 145:111678. doi: 10.1016/j.bios.2019.111678 31541787 PMC7224984

[49] Alexandrou G Moser N Mantikas KT Rodriguez-Manzano J Ali S Coombes RC. Detection of Multiple Breast Cancer ESR1 Mutations on an ISFET Based Lab-On-Chip Platform. IEEE Trans BioMed Circuits Syst (2021) 15(3):380–9. doi: 10.1109/TBCAS.2021.3094464 34214044

[50] Schrader C Schielke A Ellerbroek L Johne R. PCR Inhibitors - Occurrence, Properties and Removal. J Appl Microbiol (2012) 113(5):1014–26. doi: 10.1111/j.1365-2672.2012.05384.x 22747964

[51] Francois P Tangomo M Hibbs J Bonetti EJ Boehme CC Notomi T. Robustness of a Loop-Mediated Isothermal Amplification Reaction for Diagnostic Applications. FEMS Immunol Med Microbiol (2011) 62(1):41–8. doi: 10.1111/j.1574-695X.2011.00785.x 21276085

[52] Jakupciak JP Wang W Barker PE Srivastava S Atha DH. Analytical Validation of Telomerase Activity for Cancer Early Detection: TRAP/PCR-CE and hTERT mRNA Quantification Assay for High-Throughput Screening of Tumor Cells. J Mol Diagn (2004) 6(3):157–65. doi: 10.1016/S1525-1578(10)60506-5 PMC1867633 15269291

